# Global trends in e-labeling: a comprehensive geographical perspective

**DOI:** 10.3389/fmed.2025.1662782

**Published:** 2025-12-12

**Authors:** Agnès Dangy-Caye, Valentin Lebreton, Rebecca Lumsden, Sabine Faber, Ana Cavalli, Maria Lucia De Lucia, Sima Desai

**Affiliations:** 1 Regulatory Science and Policy Europe, Sanofi (France), Gentilly, France; 2 Global Regulatory Affairs Labeling, Sanofi US, NJ, United States; 3 Regulatory Affairs, Sanofi DE, Berlin, Germany; 4 Affiliate Regulatory Affairs, Sanofi, São Paulo, Brazil; 5 Regulatory Science and Policy International, Sanofi, Panama City, Panama

**Keywords:** e-labeling initiative, e-labeling pilot, regulation, paperless, implementation, e-labeling, electronic product information, EPI

## Abstract

**Introduction:**

This study presents a comprehensive analysis of e-labeling adoption for medicinal products worldwide. Electronic Product Information (ePI) makes available the most up to date approved medicinal product information on an electronic platform, accessed through a code or a locator included on the product packaging. Electronic Product Information can be viewable in different formats from simple ones (pdf file) to a more structured form allowing true digitalization (html/xml).

**Methods:**

The data were recovered based on internal company survey data from 182 countries collected between January 2, 2024, and April 7, 2025.

**Results:**

Based on data from 182 countries where the company operates, the findings reveal diverse implementation stages across regions, with some countries having fully incorporated e-labeling into their regulations while others explore initial pilot programs. The hybrid approach combining printed leaflets and electronic product information with some possibilities of removing the printed leaflet emerges as the predominant model in pilot studies, though most countries still mandate paper documentation. QR codes on outer packaging (74.7%) and PDF format (67.3%) represent the most widely adopted technical solutions for the implementation of e-labeling.

**Discussion:**

E-labeling offers substantial benefits including real-time information updates, environmental sustainability, and supply chain improvements. Cross-regional knowledge exchange and multi-stakeholder collaboration will be essential to accelerate harmonized global e-labeling adoption.

## Introduction

The evolution from paper-based labeling to electronic labeling (e-labeling) represents significant opportunities for the healthcare environment. For the concepts of this paper, e-labeling represents the provision of approved product information intended for patients or healthcare providers electronically (through a scannable code and/or available on a dedicated website). Various structured formats are available for ePI: e-labeling (e.g., XML/HTML or Fast Health Interoperability Resources—FHIR) as an interactive, user-friendly format offering interoperability between systems. FHIR, in particular, is defined as common standard for electronic product information, e.g., by EMA for Europe (1) and goes beyond basic digital access by supporting integration with clinical systems and electronic health records; that is where healthcare will truly begin to transform. Countries around the world are exploring e-labeling regulations, pilot programs, and/or e-labeling implementation, which aims to enhance the accessibility, accuracy, and usability of medicinal product information for patients and healthcare professionals. Additionally, the introduction of e-labeling can lead to the removal of the printed leaflet.

E-labeling initiatives and implementation offer numerous advantages, such as improved access healthcare professionals, patients, and caregivers through the internet. It has the potential to provide the most up-to-date product information available anytime, facilitating better decision-making and enhancing safety. Additionally, it promotes a better of product information patients and healthcare professionals with enhanced accessibility features (i.e., text-to-speech, searchability) and improves supply chain resilience and efficiency (1–3). Relying less on paper to deliver product information is major progress towards a more sustainable world by limiting the carbon footprint of the packaging (4) This global shift is expected to reshape communication in the healthcare industry, making current product information via e-labeling more adaptable and interactive. Ultimately, the future evolution of e-labeling may be broader, such as the provision of additional non-promotional Supplementary material (i.e., visual aids, or videos showing how to use a medicinal product) and the provision of personalized ePI that could help the patients understanding and tailor medicines information to suit their needs specifically.

A review of existing initiatives to implement e-labeling is provided in this article. Regulations regarding e-labeling and various e-labeling initiatives around the world have also been analyzed. These initiatives include implementation of e-labeling, as well as planned, ongoing and completed pilots. Operational characteristics are also presented, such as e-labeling formats available, ownership of e-labeling platforms, use of two-dimension (2D) codes (e.g., a scannable code such as a QR code) for linking to e-labeling, and statistics on countries where printed leaflets are mandatory. Understanding the global e-labeling landscape and showcasing data on e-labeling trends is critical to support key stakeholders such as Health Authorities, trade associations, legislators, pharmacy and patient groups collaborate to engage on e-labeling initiatives. This supports the adoption of digital approaches and subsequent removal of the paper leaflet especially in low-risk setting administered by healthcare professionals like hospital products and vaccines (3).

## Methods

### Study design and participants

This exploratory review is a mixed method between expert surveys conducted internally and combined with interviews of local regulatory affairs professionals working for Sanofi across the globe and cross-referencing local policies and policy makers’ statements regarding electronic product information. The research was conducted from January 2, 2024, to April 7, 2025.

Expert surveys were sent out to a total of 84 respondents selected based on their professional scope and qualifications. Respondents were selected as Subject Matters Experts that were (1) actively working in the field of regulatory affairs, regulatory science and policy and/or (2) displayed experience working on e-labeling by implementing the change of printed leaflet format locally, or through engagement with local Health Authorities and/or Industry Associations. Surveys, interviews and digital exchanges were conducted in English with authors and respondents able to converse orally and by writing.

### Expert surveys and interviews

A structured online survey tool was developed based on industry challenges and issued e-labeling policies and guidance (1, 3, 5–9). Data points were collected in the form of questions and within fields of the online survey tool enabling a full assessment for a given market to determine the stage of e-labeling discussions, initiatives and perspectives. Data points were descriptive and consisted of a combination of open-ended (free text fields) and close-ended questions (drop-down fields with predefined answers). The questionnaire was built around three angles to fully capture the country’s e-labeling situation. The first section focused on local policies and regulatory environment regarding the obligatory status of the printed leaflet and use of 2D codes on drug packaging. The second section consisted of capturing the digital and technical framework of the country with current 2D code usage, e-labeling digital platforms planned or in place and the platform owners. The third section was focused on any ongoing e-labeling pilots or initiatives. The data presented in this review were based on the data collections points and questions presented in Table 1.

**Table 1:** Data points collected in the online survey tool.

Data field	Choice of responses
Current overview	Paper; paper + isolated e-labeling; paper + e-labeling; paperless e-labeling opportunities; paperless e-labeling implemented
Is printed leaflet mandatory?	Mandatory; possible exemptions; not mandatory
Are there any regulations requiring paper labeling (paper leaflet)?	No; yes
If yes, please explain	Open field
Has paper leaflet been removed for any product in your country?	Open field
Current use of 2D codes for e-labeling	Open field
Are 2D codes already in use on any product packaging for e-labeling information?	QR code; data matrix; bar code; no code
2D code location	Outer pack; inner pack; leaflet
E-labeling format	PDF; HTML/XML; both
E-labeling platform ownership	Health authority; company; pharma association; third party; other
E-labeling initiative description	Country; region; type of initiative; status; initiative objective; initiative product criteria; dates (when possible)

The surveys were sent out by email to participants, and they were also invited to provide additional country e-labeling insight by e-mail. Upon survey introduction, an initial 30-to-60-min remote interview was conducted using videocall tools with the local experts to ensure an optimum level of engagement, purpose and understanding of the answers collected from the research.

### Data management

Answers to the survey were gathered in a central database built for the purpose of organizing information related to e-labeling, while enhancing collaboration between the researchers and participants. The regulatory environment and initiatives regarding e-labeling are constantly evolving, however this article aims to provide a snapshot of the landscape during the period of data collection. The survey instrument remained accessible and was updated until data lock on April 7, 2025.

#### Classification

**Figure 1:**
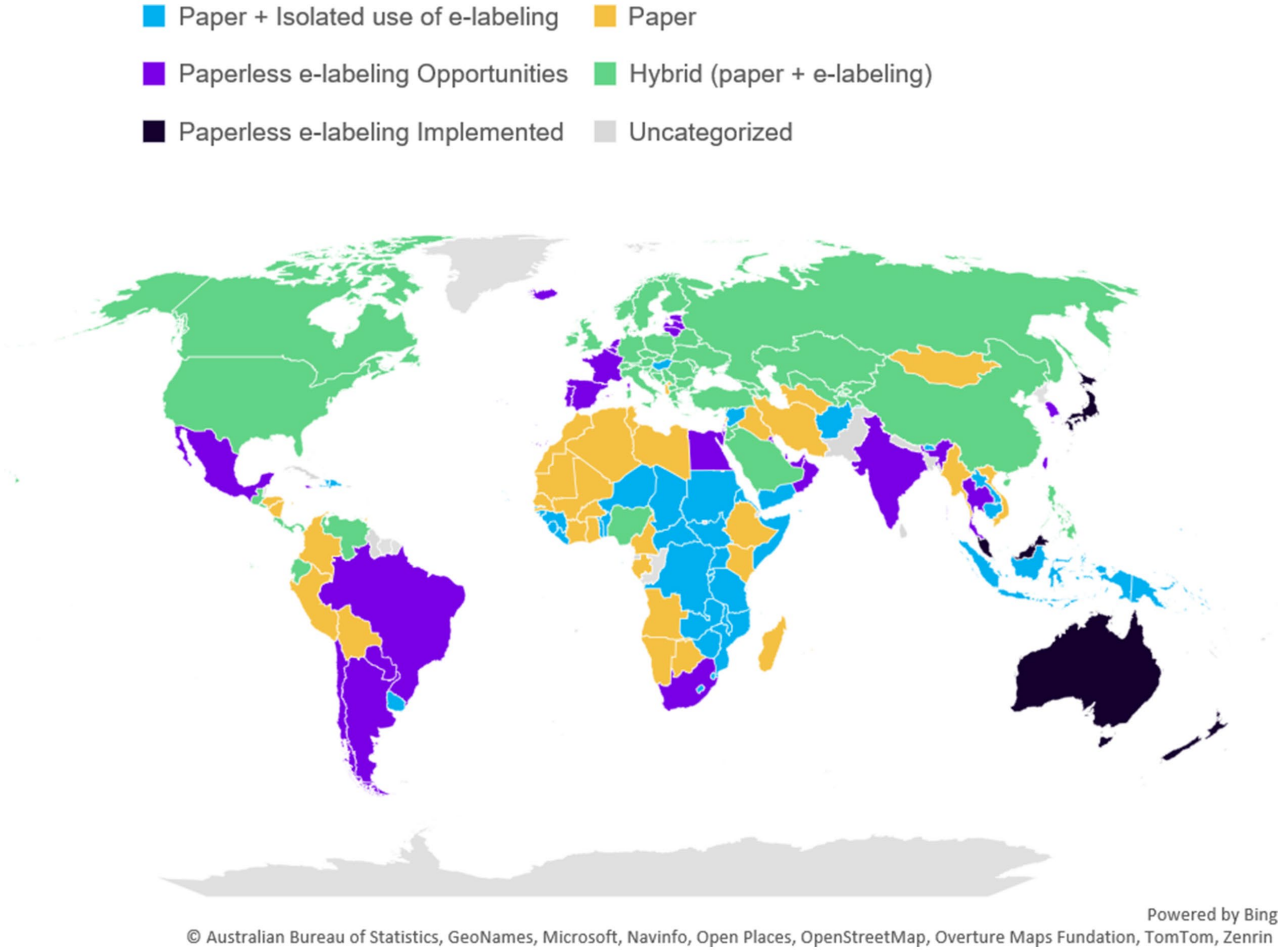
Map of country e-labeling stages across the globe as of April 7, 2025. Microsoft product screen shot(s) reprinted with permission from Microsoft Corporation.

While conducting this research and establishing mapping criteria, 5 types of classifications were established and assigned to countries based on gathered insights. The category that best represents the situation in the country, as seen in Figure 1, was selected:

It is important to note that this study focuses on human pharmaceutical products, including prescription and non-prescription drugs, and biologics. It excludes medical devices, *in-vitro* diagnostics, and other regulated health products, as Regulatory requirements for labeling can differ between product classes.

##### Paperless e-labeling implemented

This category indicates that the country or region has transitioned to using e-labeling at least for most medicinal products without the provision of an accompanied paper leaflet. All product information, previously in the form of printed leaflets, is provided digitally. This approach maximizes accessibility through mobile devices or online platforms, ensuring that users always have the most current information without the environmental impact of a required printed leaflets.

##### Paperless e-labeling opportunities

In this scenario, both paper-based and digital product information options are available. While traditional printed leaflets are still in use, there are opportunities to remove it from the commercial packaging for selected products or product categories. This means that paperless packaging is being piloted for products used in specific healthcare settings (such as vaccines, injectables, and other parenteral products administered by healthcare professionals in hospitals or clinical settings), where printed leaflets may be removed or for specific range of products based on exemptions of legal obligations, including certain patient-administered prescription or OTC drugs.

##### Hybrid (paper + e-labeling)

This category refers to a situation where printed leaflets are still widely used alongside e-labeling. The digital version is intended to complement the traditional paper approach. Patients or healthcare professionals can access information in their preferred format, reflecting an environment that is gradually adapting to digital solutions but maintains strong ties to the traditional method.

##### Paper + isolated e-labeling

This category refers to a situation where printed leaflets are still the main form of dissemination of product information, but e-labeling is possible for a limited number of products (e.g., humanitarian health programs).

##### Paper

This category indicates that all product information is provided exclusively in paper format, with no electronic alternatives available.

## Results

### A global snapshot of e-labeling

#### Current overview of the regulations—country perspective

The initial intent of this research was to assess the regulatory landscape in countries and regions to determine the potential acceptability of e-labeling initiatives and implementation. The global dataset encompassing 182 countries where the company operates, the findings identifying the various types and stages of e-labeling framework indicate the potential to assign categories (cf. above Classification) to countries to summarize their general state and readiness for e-labeling. This includes evaluating their existing digital infrastructure and regulatory framework.

**Figure 2:**
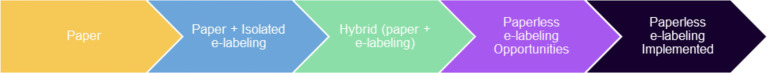
E-labeling adoption scale observed in countries around the world.

We have collected information from a total of 182 countries. Figure 1 provides an overview of e-labeling regulatory environments, and country readiness for e-labeling implementation. This figure highlights the varying stages of e-labeling progress and adoption across different countries providing a broader global view based on the categories established in Figure 2. Categorization of countries are provided in Table 2.

**Table 2:** General classification of countries after regulatory assessment of e-labeling adoption of 182 countries.

Category of assessment	Total countries *n* = 182	(%) (100)
Paperless e-labeling implemented	6	(3)
Paperless e-labeling opportunities	29	(16)
Hybrid (paper + e-labeling)	52	(29)
Paper + isolated e-labeling	44	(24)
Paper	37	(20)
Uncategorized^a^	14	(8)

a Countries or territories were designated as “Uncategorized” when collected data regarding current label regulation and e-labeling were considered insufficient or inapplicable (unregulated markets).

Evaluating all countries, a total 48% (*n* = 87) are actively engaged in some form of e--labeling as a standard practice in their market [*Hybrid model (paper + e-labeling), paperless e-labeling opportunities/implemented*]. Among them, 19% (*n* = 35) are considered open to a paperless approach either as part of pilots and/or transitory regulation [Paperless e-labeling Opportunities—16% (*n* = 29)] or as an established approach [Paperless e-labeling Implemented—3% (*n* = 6)]. These countries are leveraging e-labeling in replacement of paper product information in local language in specific categories of medicinal products.

**Figure 3:**
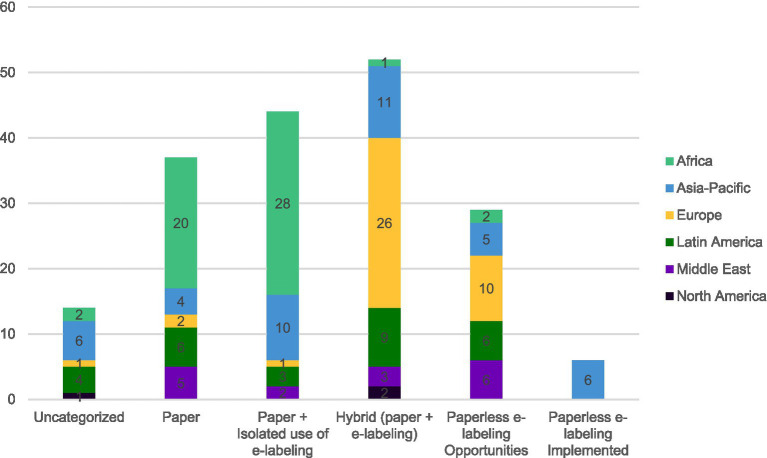
Regional distribution of countries per e-labeling adoption phase based on 182 countries.

The data collected also allows the identification of trends among key regulatory regions around the world. Figure 3 provides an overview of the categories described earlier by region to identify if any regions play a leading role in the e-labeling evolution. Countries across all regions are in different stages of e-labeling adoption.

As seen on Figure 3, 22% (*n* = 37) of countries are considered “*Paper*” while 78% (*n* = 131) are leveraging e-labeling in some capacity. While 26% (*n* = 44) of countries demonstrate isolated e-labeling distribution possibilities, predominantly in African countries. Meanwhile, in a third (31%, *n* = 52) of countries, the product information is found in both formats in parallel, with most European countries appearing here. Paperless opportunities seen in 17% (*n* = 29) of countries are seen across all regions. However, Asia-Pacific is the only region where advanced e-labeling is seen (4%, *n* = 6). In these countries e-labeling and removal of the paper product information have been implemented (“*e-labeling implemented*”) in law and/or regulations and have become a standard practice beyond pilots or hybrid phases for most products.

### Printed leaflet regulation

#### General overview

The survey sent to affiliates included a question regarding if providing the printed leaflet is mandatory in the local regulation and/or guidance for human pharmaceutical products, including prescription and non-prescription drugs, and biologics (as defined in the Methods section—Data Management). Requirements for medical devices, *in-vitro* diagnostics, and other regulated health products are not included in this analysis.

**Figure 4:**
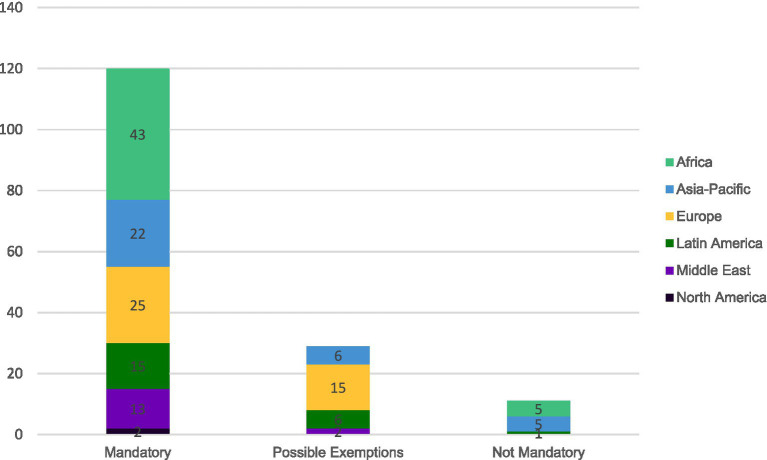
Regional distribution of regulatory requirements for printed product information in medicinal product packaging (*n* = 160 countries; prescription, non-prescription drugs, and biologics).

Answers from 160 countries were received, identifying that in 75% (*n* = 120) countries, printed leaflets were required, while 18% (*n* = 29) countries allowed for possible exemptions for this requirement and 7% (*n* = 11) countries did not require the printed leaflet. A breakdown of this data by regions is summarized in Figure 4.

Figure 4 shows that the use of printed leaflets remains mandatory as a routine supply for most countries on all continents for the medicinal product categories included in this study. Countries considering paper product information as not mandatory are found in Africa and Asia-Pacific.

#### Use of 2D codes in e-labeling

The next step was to understand the regulations in countries regarding the use of 2D codes on product packaging. Among the 71 responding countries, 75% of them (*n* = 53) are already using a 2D code for e-labeling purposes.

**Figure 5:**
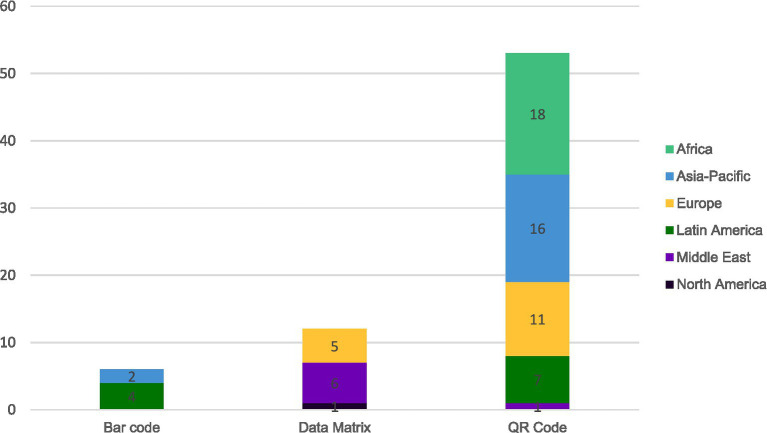
Type of code for countries using 2D codes for e-labeling purposes based on 71 responding countries.

Further analysis was performed to determine the type of 2D code used when 2D codes were permitted, required or not mentioned in the regulations (see Figure 5).

Figure 5 shows that QR codes are the main type of 2D code used currently (75%; *n* = 53) followed by Data-matrix codes (17%; *n* = 12). Barcodes are used in some countries (8%; *n* = 6). The various two-dimensional codes for electronic labeling present different advantages and challenges to implement (10), and the results of these factors are illustrated here. The wide adoption of QR code over the other 2D for e-labeling is linked to the fact that most modern smartphones come with native QR code scanning capabilities integrated to the camera apps. Patients and healthcare professionals are then not required to install a dedicated app. This makes QR code a versatile tool for digital information sharing, with widespread compatibility across mobile manufacturers and regions.

In Europe, almost all countries have a Data Matrix code (“serialization code”) printed on prescription products and some over the counter (non-prescription) products on the outer box, as legally requested by the Falsified Medicines Directive (11). This Data Matrix code can be used for linking to e-labeling, e.g., via a downloadable app, which is already in place in Germany (12) and in Nordic countries (13–15). Barcodes scanned via apps provide an additional option, that is used, e.g., in Germany for linking to e-labeling for over the counter (OTC) products (not carrying a “serialization code” as not required by Falsified Medicines Directive). The possibility and acceptance by authorities to use such codes for linking to e-labeling has existed for many years and is described in European regulatory guidance documents (16).

**Figure 6:**
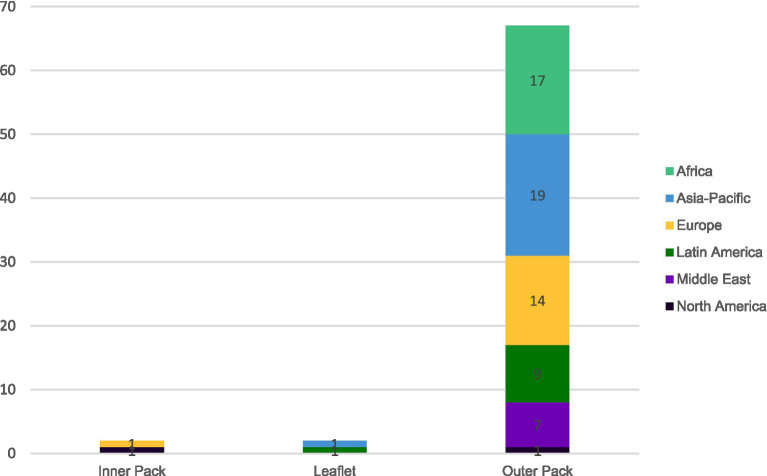
Type of 2D code location in current practice based on 71 responding countries.

Based on the 71 survey respondents, the 2D code to reach the e-labeling version can be found either in the outer pack, on the printed leaflet or in the inner pack. Figure 6 shows the distribution in current practice.

Figure 6 shows that the 2D code is most often found on the outer packaging (93%; *n* = 67) across all regions. The 2D code is printed on the leaflet in 2 countries, mainland China and Mexico. The 2D code is printed in the inner pack in Spain and US however US will also routinely accept that the 2D code be printed on the outer pack. For some countries, a 2D code may be available in a secondary location, however, this represents the commonly reported location.

#### Format of e-labeling

**Figure 7:**
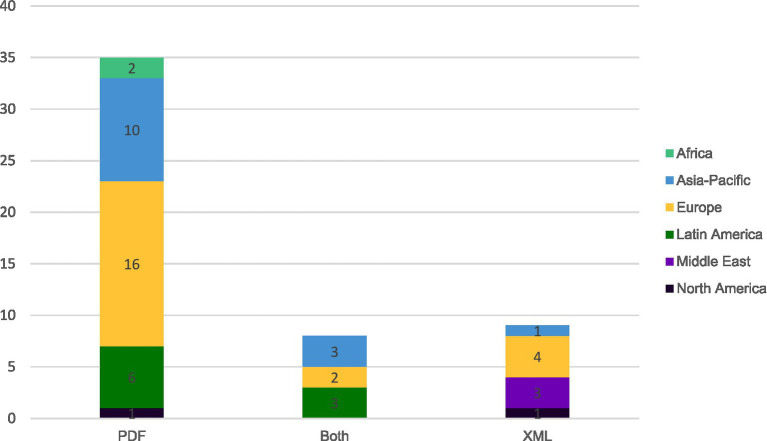
Format of e-labeling in current practice in 52 responding countries.

The format of e-labeling information can be either in PDF or in XML/HTML format. While XML represents a structured data format designed primarily for platform-independent data exchange, and HTML functions specifically as a markup language for creating web content, both can be categorized as digital content formats. This digital classification distinguishes them from paper-oriented document formats like PDF, which was originally designed to preserve document fidelity across different systems while maintaining a print-ready presentation. Data from 52 countries was received and results are presented in Figure 7 below.

In 67% of countries (*n* = 35), e-labeling is available in PDF format, while XML/HTML format is used in 17% of countries (*n* = 9). In 15% (*n* = 8) of the countries, both formats are used.

The formats are variable across all regions except in Africa where PDF format is preferred and in the Middle East where XML/HTML format is used.

#### Platform ownership

**Figure 8:**
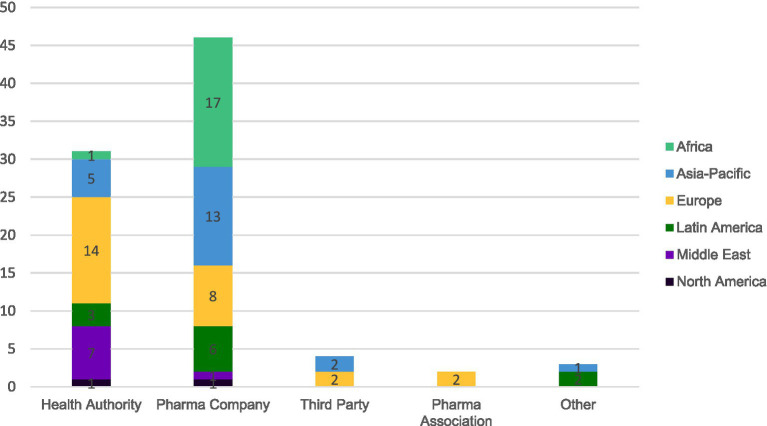
Ownership of e-labeling platforms for 86 responding countries.

Product Information distributed via e-labeling is managed on a digital platform, with variable ownership of these platforms. Figure 8 identifies the distribution of ownership of the platforms currently in use based on 86 survey respondents.

Figure 8 shows most e-labeling platforms are owned by pharmaceutical companies (53%; *n* = 46) or by Health Authorities (36%; *n* = 31). Third parties can also own the platform, hosting e-labeling in 5% of countries (*n* = 4), in Europe (i.e., eMC for the UK, GI 4.0^®^ for Germany, Felleskatalogen for Norway) (14, 15, 17) and Asia-Pacific.

#### Overview of pilots

**Figure 9:**
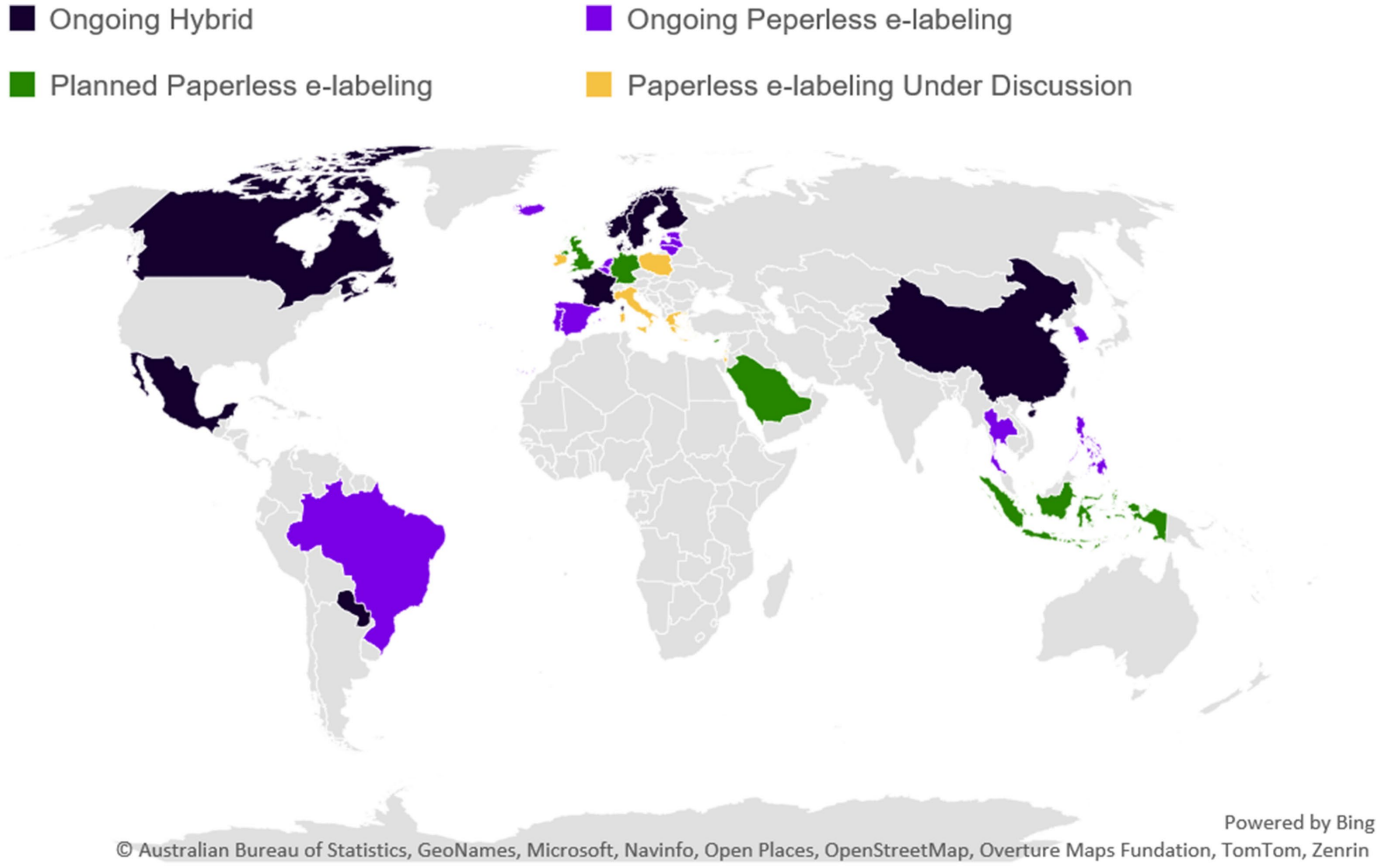
E-labeling pilots around the world. Ongoing paperless labeling (*n* = 13), planned paperless e-labeling (*n* = 5), paperless e-labeling under discussion (*n* = 5), ongoing hybrid (*n* = 13). Microsoft product screen shot(s) reprinted with permission from Microsoft Corporation.

Figure 9 provides a global view of e-labeling pilots from various perspectives identifying where they are under discussion, planned, or currently ongoing as well as the scope of intent related to inclusion of digital access to product information with or without printed leaflet removal.

Supplementary material contain a regional review with additional qualitative information recovered from the survey and interviews.

### Research limitations

The data presented in this article is a representation of our internal survey, interviews and digital exchanges with regulatory affairs personnel at the regional and affiliate level at Sanofi, and cross checked with online sources where possible from January 2, 2024, to April 7, 2025.

A key limitation of this study is that the data comes solely from an internal survey and operational experience. This single-company perspective introduces potential bias, as product portfolio, market priorities, therapeutic focus areas, and regulatory interactions may not be fully representative of the entire pharmaceutical industry. Companies with different product mixes (e.g., those focused primarily on OTC products, generic manufacturers, or specialty pharmaceutical companies) may have different experiences with e-labeling regulations and implementation. Additionally, the company’s strategic priorities and market presence in certain regions may influence the depth and focus of regulatory intelligence gathered. Readers should interpret the findings as reflective of one major pharmaceutical company’s experience rather than a comprehensive industry-wide assessment.

The primary data source for this study is self-reported information from the company regulatory affairs personnel. While these individuals are subject matter experts with direct knowledge of local regulatory requirements, this approach carries inherent limitations. Potential perception bias, variations in interpretation of regulatory requirements across different affiliates, and incomplete information due to rapidly evolving regulations may affect data accuracy. Although information were cross-checked with publicly available sources where possible, independent verification of all data points was not feasible.

While the analysis covers 182 countries, representing a substantial portion of global pharmaceutical markets, we recognize that regulatory landscapes continue to evolve, and our findings represent a snapshot based on the company’s operational experience rather than an exhaustive global assessment.

A significant limitation is that this study does not systematically disaggregate data by product type (e.g., prescription drugs, OTC products, biologics, vaccines, parenteral products). Regulatory requirements and e-labeling implementation timelines can differ substantially between these categories. For example, Figure 4 on printed leaflet requirements presents aggregated data across all pharmaceutical product types in the company’s portfolio, which may mask important variations in requirements for different product categories within individual countries.

For simplification purposes, we have established five categories to classify countries to give an overview of e-labeling. These categories provide an overall view of where they are in the e-labeling journey. They do not consider specifics for each country such as the scale of e-labeling usage in the country, scope of products, consumer behavior and public sentiment.

The classification system is inherently subjective, as it requires interpretation of regulatory frameworks that may be ambiguous or in transition. To enhance rigor and minimize individual bias, country classifications were assigned through a collaborative process involving multiple regulatory affairs experts from different regions. In cases where initial classifications differed, the team engaged in structured discussion to reach consensus, considering factors such as regulatory text interpretation, and feedback from local affiliates. Despite these efforts to ensure consistency, a degree of subjectivity remains. The subjectivity of this classification is representative of the need for common standards in e-labeling regulation and approach to implementation.

## Discussion

### Current view of e-labeling adoption

The data presented in this article show the heterogenicity in the use of e-labeling worldwide. Although e-labeling is not necessarily formally introduced into the legal or regulatory framework of many countries, the various statuses of e-labeling implementation suggest a global trend to move towards e-labeling adoption. Many countries are already involved in e-labeling initiatives either through pilots, as hybrid or paperless approaches, or allow e-labeling implementation (either in full or for certain categories of medicines). The data and visual representations of the current e-labeling approaches and factors underscore the diverse regulatory and digital tools being used worldwide, as countries transition from traditional methods to more accessible and efficient digital solutions.

Indeed, the current view of e-labeling practices shows that only a minority of countries (3%), in Asia-Pacific, have fully implemented e-labeling within their legal and regulatory framework for most products. While 20% of countries are still requiring only printed leaflets, a large number of countries (45%) are open to options including hybrid “paper + e-labeling” or paperless opportunities. This demonstrates a clear trend to move towards electronic opportunities in a world adopting more sustainable practices adapted to environmental concerns, access to real-time product information and improved user communications.

The analysis of the 182 countries reflects the different status of e-labeling implementation regionally. Additional information is included in Supplementary material below for all regions.

*Europe*: Most countries in Europe are in a hybrid situation. The printed leaflet still remains mandatory under European Union law but with current revision of the European legislation, the e-labeling concept is being introduced in the draft proposals (currently in negotiation). Independent of the future legal framework, EMA has defined key principles for the introduction of e-labeling (1) in a common standard, the concept of which has just been tested in EMA’s e-labeling pilot (18) and is being further developed for a proposed stepwise implementation of e-labeling starting with Centrally Authorized Products. In parallel, we are seeing more National Competent Authorities (NCAs) launching pilots to test e-labeling and remove the printed leaflet under specific conditions.

*Latin America*: Latin American countries are making significant strides in implementing e-labeling for medicinal products, with varying degrees of progress across the region.

Some countries in the Latin American region allow both digital and printed formats focusing on understanding the country’s needs, both from a regulatory perspective and technology infrastructure required to support e-labeling.

*Asia-Pacific*: E-labeling implementation varies significantly across Asia-Pacific. Some countries already have comprehensive guidelines (Australia, New Zealand, Malaysia, Singapore, Thailand, Indonesia, South Korea, Taiwan (ROC), mainland China, Japan) with possibilities to provide products with or without printed leaflets by using QR code or GS1 barcode. In some countries, the labeling is already available in online information systems. Pilots are already implemented or encouraged in these countries but also in countries not yet having official regulation in force. Pilots are, in general, for some specific product types and with possibilities to remove the leaflet. Other countries have not yet started to change or implement their regulation or initiate pilots, but they can be open to discuss case by case implementation (Vietnam, Philippines, India, Brunei). All those initiatives allow a step forward in the adoption of e-labeling (19).

*North America*: In the United States, a hybrid approach is adopted using QR code and links to online searchable databases, however printed product information remains mandatory under the U.S. regulations. U.S. is continuing to explore options to further modernize product information and ensure that most current prescribing information is publicly and easily accessible.

In Canada, printed leaflets remain mandatory for prescription drugs, though exemptions are granted case-by-case for some products, but regulatory authorities worry that eliminating printed leaflets may limit information accessibility, especially in remote Canadian areas.

*Middle East*: Egypt is exploring e-labeling with projects following both a three-stage implementation process for medicinal products, and a two-stage approach with similar criteria for biologics with QR code ensuring direct access to EDA (Egyptian Drug Authority) hosted information.

- Stage 1 (began February 2022) introduced voluntary dual-format labeling with QR codes alongside printed leaflets.
- Stage 2 (current) allows gradual removal of printed leaflets for hospital-administered products.
- Stage 3 will expand e-labeling to more products with standardized digital formats.

For biologics, implementation follows a two-stage approach with similar criteria focusing on hospital-use products and vaccines, with specific QR code requirements ensuring direct access to EDA-hosted information. Both Saudi Arabia and United Arab Emirates (UAE) accept hybrid e-labeling, however, a paper leaflet is still required.

The Saudi Food and Drug Authority (SFDA) developed the Tammeni app, which allows users to scan barcodes on food, drugs, cosmetics, and medical devices to access verified product information. It is linked to the Saudi Drugs Information System (SDI), which hosts digital leaflets and product data for all registered medicines.

Jordan FDA is implementing mandatory e-labeling using GS1 Data Matrix 2D barcodes on packaging, while maintaining printed leaflets in this phase. The system uses GTIN and batch number from the Data Matrix (also containing expiry date and serial number) to link to XML-format electronic leaflets. Patients can access these leaflets through a dedicated mobile app developed by Jordan FDA, which features an XML reader that converts the information to HTML for easy reading (20).

*Africa*: Pharmaceutical packaging across Africa remains predominantly paper based. However, initial steps towards digital transformation include successful QR code implementation in several countries, which are supported by a few official guidelines. Ongoing pilot programs are expected to significantly shape future e-labeling strategies across the continent.

In South Africa, SAHPRA permits the use of QR codes on packaging; however, the paper leaflet is still required to remain inside the pack.

The results clearly demonstrate that all regions are on the pass to e-labeling, including for some, the complete removal of the printed leaflet. This provides an opportunity for both global and regional sharing amongst Health Authorities, allowing them to share their experiences and lessons learned on their implementation pathway to e-labeling. In particular, information exchange on how they are managing digital access to product information for patients could support quicker implementation of e--labeling practices globally. E-Labeling would be a suitable topic for discussion and action by multi-national organizations such as the International Pharmaceutical Regulators Programme (IPRP) or continued discussions at the International Conference of Drug Regulatory Authorities (ICDRA) to leverage these learnings into recommendation and/or guidance (21).

Critically, feedback and observations from the many ongoing pilots globally are essential to create a clear vision of stakeholder perspectives and to drive next steps, whether full paperless e-labeling adoption or a hybrid approach. We’re encouraged to see many markets focusing on paperless e-labeling pilots in their planning and under discussion stages, indicating a global shift towards eliminating printed leaflets. Initial e-labeling initiatives, whether in pilot design or optional/limited implementation, appear to influence country’s confidence with digital solution adoption.

Observationally most ongoing paperless e-labeling pilots have begun with medicines primarily administered by Health Care Professionals or in healthcare settings before including medicines provided directly to patients. However, as e-labeling approaches and learnings can be shared between health authorities and/or stakeholders across regions, it may be possible for markets to move to implementation of paperless e-labeling in the absence of an initial pilot. Thus, initial design of the e-labeling initiative in a given market should be well intentioned with the future desired outcome of implementation in mind.

We also note that digital e-labeling approaches can vary. Based on our survey results, in terms of 2D code use, Data Matrix or QR Codes on outer packaging seem to be preferred options for linking to e-labeling.

The structured data format of e-labeling (with, e.g., XML/HTML or FHIR) is an interactive, user-friendly format offering interoperability between systems. Web compatible formats of the product information become key as they feature device versatility and high interoperability between systems. Meanwhile, the variation in platform ownership in each region/country warrants additional discussions to converge on approaches that assure patients and Health Care Professionals have the latest version of the product information as rapidly as possible, while also addressing regulatory operational challenges such verification of version management, across different platforms.

Paperless evolution: Providing the printed leaflet in the packaging of medicinal products is a traditional practice in the industry, in many countries, and routinely seen as mandatory as interpreted by law or regulation. However, across the 182 countries included in the analysis, the results do identify instances where paper is actively being removed for some product types, either at the request of the pharmaceutical company (possible by exemption procedures in some markets) or health authority.

Stakeholders’ concern for the complete removal of paper is access to e--labeling for those with minimal internet access, or digital literacy (22). Trade associations and working groups, including representatives from the patient representatives, HCP associations and the industry are working to find possible solutions to provide a print-out to patients in need in their respective countries/regions. An option which is already established in countries like Japan and Australia, is to deliver the printouts to the patients in pharmacies.

Moreover, it can be expected that the percentage of the population requiring paper version of the product information may decrease over time as internet access, usage of smartphones and digital literacy increase. Education campaigns should be introduced to aid this progression.

Like any change to long-standing practices and/or process, a complete replacement of paper by e-labeling will take time to accept and adopt due to the required change in mindset, legislative framework and/or regulation/guidance. Paperless e-labeling initiatives can be effective towards, gaining experience, confidence and preparing countries for a more future oriented digital approach. Additionally, they provide valuable opportunities to gain practical experience with boxes free of paper leaflets. This includes exploring potential benefits such as reduced packaging sizes, while simultaneously addressing technical considerations like ensuring the stability of medicinal products within the packaging when paper leaflets are removed. In any case, the shift from paper to paperless e-labeling should be considered with an approach taking into consideration the local legal framework, regulatory guidance, health authority views, patient advocacy groups, environmental impact and overall business value.

We imagine the scenario where a patient can easily access real-time product information at their fingertip for their consumption in a way they can easily engage with, in a more environmentally sustainable way (2, 23, 24). Building on the results of this paper we would propose the following four areas of prioritized activity to enable the shift from paper to e-labeling implementation and perhaps to a truly paperless system globally:

1) *Communication and collaboration with all stakeholders nationally and/or regionally (where packaging is shared)*: Engagement with key stakeholders (patient advocacy groups, trade associations, legislators, health authorities and health care professionals) within each country is a critical enabler for successful implementation of e-labeling approaches. Stakeholders should work together to address e-labeling barriers, while leveraging learnings from experienced global e-labeling infrastructures to showcase and educate on e-labeling benefits.
2) *Knowledge sharing of global e-labeling approaches*: Labeling is often seen as a national competence. However, Health Authorities should share e-labeling learnings and approaches through global regulatory organizations such as IPRP & ICDRA to facilitate e-labeling adoption through regulatory framework and digital technologies. In general, countries with advanced ePI implementation should share their knowledge, experiences, and best practices from different stakeholder groups to support other regions in their digital transformation journey.
3) *Implementation of e-labeling pilots*: Pilots can be an effective step towards gaining experience, building confidence and preparing countries for a more future oriented digital approach. Expanding beyond national pilots to global e-labeling pilots should be explored such as testing if e-labeling can be used to support globally supplied critical medicines, i.e., WHO Prequalified vaccines and/or products on the WHO Essential medicines list.
4) *Focus on future-proofing regulatory & legal frameworks*: With the advent of Artificial Intelligence/Machine Learning technologies it will be essential that any mandated frameworks on product information are future-proofed to enable rapid adoption of emerging digital technologies. Frameworks should prioritize describing the required content/information to be delivered and/or available to healthcare professionals and patients and not the mechanism of delivery—whether paper or digital tools.

## Data Availability

The raw data underlying the conclusions of this article are available from the corresponding author upon reasonable request.
