# Supplementary material for: Global trends in e-labeling: a comprehensive geographical perspective
Source: Front Med (Lausanne). 2025 Dec 12;12:1662782. doi: 10.3389/fmed.2025.1662782 (PMC12740925; doi:10.3389/fmed.2025.1662782)
Supplement: Supplementary file 1 [file Data_Sheet_1.docx]

Supplementary Information:

**Europe**

Health Authorities are launching pilots in which pharmaceutical companies can volunteer to participate and remove the printed leaflet under specific conditions. These pilots are mainly running in hospital settings, based on exemptions given for specific products for a specific time, e.g. Belgium, Luxemburg, Spain, Iceland, Portugal, the Netherlands, France and the Baltics. There are additional pilots planned, under discussion, or started to extend the scope beyond the hospital settings. For example, in the Baltics, vaccines are also included in the ongoing e-labeling pilot. An integration of e-labeling into health applications is also currently in planning stage (e.g. in Germany for health insurance apps). Furthermore, exemptions from the obligation to provide printed leaflets in local language have been granted by National Competent Authorities in certain circumstances, e.g. in case of shortage risks for specific drugs. In the Nordic countries, beginning early 2025, a planned pilot project introduces English-language common packs with leaflets in local language provided as electronic leaflet in the 5 Nordic countries.

There are further countries that have started discussions about e-labeling pilot initiatives. In the UK, an ePI Task Force has been set up - a multi-stakeholder consortium comprised of a variety of stakeholders including multiple trade associations as well as big and small pharmaceutical companies. The Task Force is in communication with the MHRA regarding the initiation of an e-labeling pilot which would aim to add a 2D barcode to packs and remove the printed leaflet. The medicinal products for inclusion in the pilot are still under discussion.

**Latin America**

Latin American countries are making significant strides in implementing e-labeling for medicinal products, with varying degrees of progress across the region.

Brazil has recently advanced in the implementation of paperless e-labeling through the approval of an optional pilot project by ANVISA. Initially, the e-labeling will be implemented for a limited group of medicines, which includes free samples, medicines intended for healthcare facilities (except pharmacies and drugstores), government destination, and OTC medicines sold in multipacks. These medicines must have been available in the national market for at least five years. The project will be in force until December 31, 2026. The information collected and monitored during this period should serve as a subsidy for the future definitive regulation and potential scope expansions.

Argentina is also advancing rapidly. In January 2025, ANMAT released a public consultation for e-labeling implementation and are working to further enhance their proposal.

Paraguay has a regulation in place for e-labeling. The QR code may replace the physical leaflet authorized by the health authority for most medicinal products, excluding those identified as high-risk in a specific list for which the printed leaflet may remain. El Salvador would agree to evaluate the e-labeling on innovative products.

Mexico's regulations don't explicitly prohibit QR codes or digital leaflets, which identifies opportunities to first implement e-labeling with QR codes while maintaining printed leaflets.

All the three following countries are moving toward e-labeling systems while maintaining printed information requirements. In Colombia, the Ministry of Health is developing new labeling guidelines that include e-labeling alongside printed information in the initial stage. While in Ecuador, the reformed regulations for small molecules and biological medicines now include optional e-labeling in addition to printed information. The authority is developing a platform to store approved product information.

**Asia Pacific**

E-labeling implementation varies significantly across Southeast Asia. Malaysia and Singapore lead the region with comprehensive guidelines introduced in 2021 and 2023, respectively, that allow voluntary paperless distribution of pharmaceutical products. Both countries permit manufacturers to replace traditional printed leaflets with digital information accessible via QR codes on packaging, resulting in environmental benefits while maintaining consumer access to essential medicinal information. The Pharmaceutical Industry Trade Association collaborated closely with Health Authorities in both countries to facilitate this transition.

Thailand, Indonesia, South Korea, Taiwan (ROC), mainland China, and Japan have also established e‑labeling guidelines, though implementation differs. Indonesia, Japan, South Korea, and Taiwan (ROC) can accept paperless product information, while Indonesia has recently initiated a pilot program.

In mainland China, the hybrid (Paper PI + e-labeling) approach is ongoing with the NMPA e-labeling “Age-appropriate and Barrier-free” pilot implementation since October 2023. The pilot scope is only applicable to oral and topical medicinal products. In the Chinese pilot, full version (Printed leaflets and e-labeling) and (simplified printed leaflets and full version e-labeling) are allowed. A 2D (e.g. QR code) should be added onto the printed leaflet. With no current timeline to end the pilot.

Japan's 2019 amendment to its pharmaceutical regulations mandated digital package inserts for pharmaceuticals and medical devices (except OTC products), allowing information access via GS1 barcode scanning on medication and device packaging, with elimination of printed leaflets by July 2023. This health authority led transition from the previous requirement of bundled printed leaflets offers benefits including access to current safety information and environmental sustainability, while integrating with PMDA's online information system where product information was already being published.

South Korea's MFDS expanded its e-labeling pilot in early 2024 to include 109 hospital-use injectable products from 27 companies, allowing QR code implementation with or without printed leaflets. This digital approach enhances information accessibility and environmental sustainability, though the program will maintain its current scope through 2025 with no expansion plans beyond existing participants.

Vietnam has incorporated e-labeling information in guidelines but hasn't enforced implementation, and the Philippines may resume discussions in the coming year. Despite lacking formal guidelines, health authorities in India and Brunei remain open to case-by-case e-labeling implementation, with Brunei showing flexibility due to medication supply connections with Malaysia and Singapore, while India still requires printed leaflets but may consider QR codes for digital access.

In New Zealand provision of a printed patient information leaflet is not mandated and accessing medicines information from the health authority website (Medsafe) is well established.

Australia's regulatory framework permits electronic distribution of patient information through QR codes or URLs on packs as an alternative to printed leaflets. However, for self-administered injectables, unless an exemption is obtained, a specific condition of registration requires printed prescribing information to be included in the pack. In addition, for products requiring preparation before use, printed instructions are required in the package in case of insufficient space to include this information on the package. Australia is planning consultations that will provide opportunities to advocate for the complete removal of printed leaflet.

**North America**

U.S. Food and Drug Administration (FDA) has an online searchable database (Drugs@FDA) which includes drug product information including patient information, labels, approval letters, reviews and other product information. FDA also has a specific online label repository (FDALABELS) where customizable searches of over 140,000 labeling for human prescriptions drug, non-prescription drugs and other products can be found. In the U.S., to ensure accessible prescribing information and enhancing patient safety, sponsors can voluntarily include a scannable Quick Response (QR) code in product labeling. These approaches make the product information electronically available for healthcare providers, patients and caregivers. The U.S. is continuing to explore options to further modernize product information, however currently, removal of the paper labeling component in packaging is not possible.

In Canada, exemptions for printed leaflet removal are granted case-by-case for products sold in bulk/repacked at pharmacy level or administered by healthcare professionals. QR codes on specific products have been approved as an initial step towards e-labeling. Canadian Regulation C.04.019 requires biologic drugs to display dosage and directions on both inner and outer labels, with physical inserts providing comprehensive safety information when labels cannot. Manufacturers seeking paperless alternatives must explain how patients will access essential safety information at dispensing time, with final decisions at Health Canada's discretion.

**Middle East**

Egypt's e-labeling project follows a three-stage implementation process for medicinal products, with a parallel approach for biologics. For pharmaceuticals: Stage 1 (began February 2022) introduced voluntary dual-format labeling with QR codes alongside printed leaflets; Stage 2 (current) allows gradual removal of printed leaflets for hospital-administered products; Stage 3 will expand e-labeling to more products with standardized digital formats. For biologics, implementation follows a two-stage approach with similar criteria focusing on hospital-use products and vaccines, with specific QR code requirements ensuring direct access to EDA-hosted information.

Turkey will implement a hybrid e-labeling approach (printed leaflet + e-labeling) effective January 1, 2026. Products administered by health care professionals (such as vaccines, radiopharmaceuticals, products administered in hospitals), are exempt from the e-labeling requirement. For all other medicinal products, regulations mandate providing both electronic Patient Information Leaflets and printed instructions together in the packaging. Currently the Turkish regulations specifically prohibit using electronic Patient Information Leaflets as a complete replacement for printed Patient Information Leaflets.

Jordan FDA is implementing mandatory e-labeling using GS1 Data Matrix 2D barcodes on packaging, while maintaining printed leaflets in this phase. The system uses GTIN and batch number from the Data Matrix (also containing expiry date and serial number) to link to XML-format electronic leaflets. Patients can access these leaflets through a dedicated mobile app developed by Jordan FDA, which features an XML reader that converts the information to HTML for easy reading.

**Africa**

Across Africa, pharmaceutical packaging primarily relies on paper-based inserts, though a digital transition is gradually emerging. While implementing dual systems with both paper and QR codes requires individual negotiations with each country's Health Authority, several Global Health Unit-managed nations including Tanzania, Rwanda, Zambia, and Benin have already successfully adopted QR codes. South Africa stands at the forefront with official guidelines permitting QR codes and ongoing pilots exploring the complete elimination of printed leaflets. The outcomes of these pilot programs will significantly influence future implementation strategies throughout the continent, reflecting the varying regulatory landscapes across African nations.

The list below provides the countries evaluated in this review and represented in the figures provided in this paper, and the regions they were attributed to.

**Asia-Pacific (42 countries)**

Afghanistan, Armenia, Australia, Azerbaijan, Bangladesh, Belarus, Bhutan, Brunei, Cambodia, China, Georgia, Hong-Kong, India, Indonesia, Japan, Kazakhstan, Kyrgyzstan, Lao People's Democratic Republic, Macao, Malaysia, Maldives, Micronesia, Mongolia, Myanmar, Nepal, New Zealand, Pakistan, Papua New Guinea, Philippines, Russia, Singapore, Solomon Islands, South Korea, Sri Lanka, Taiwan, Tajikistan, Thailand, Timor-Leste, Turkmenistan, Tuvalu, Uzbekistan, Viet Nam

**Europe (40 countries)**

Albania, Austria, Belgium, Bosnia and Herzegovina, Bulgaria, Croatia, Cyprus, Czech Republic, Denmark, Estonia, Finland, France, Germany, Greece, Hungary, Iceland, Ireland, Italy, Kosovo, Latvia, Liechtenstein, Lithuania, Luxembourg, Malta, Moldova, Montenegro, Netherlands, North Macedonia, Norway, Poland, Portugal, Romania, Serbia, Slovakia, Slovenia, Spain, Sweden, Switzerland, Ukraine, United Kingdom

**Africa (53 countries)**

Algeria, Angola, Benin, Botswana, Burkina Faso, Burundi, Cameroon, Cape Verde, Central African Republic, Chad, Comoros, Democratic Republic of the Congo, Djibouti, Egypt, Equatorial Guinea, Eritrea, Ethiopia, Gabon, Gambia, Ghana, Guinea, Guinea-Bissau, Ivory Coast, Kenya, Lesotho, Liberia, Libya, Madagascar, Malawi, Mali, Mauritania, Mauritius, Morocco, Mozambique, Namibia, Niger, Nigeria, Rwanda, Sao Tome, Senegal, Sierra Leone, Somalia, South Africa, South Sudan, Sudan, Swaziland, Tanzania, Togo, Tunisia, Uganda, Zambia, Zanzibar, Zimbabwe

**Latin America (28 countries)**

Argentina, Aruba, Bolivia, Brazil, Chile, Colombia, Costa Rica, Cuba, Curacao, Dominican Republic, Ecuador, El Salvador, Guatemala, Guyana, Haiti, Honduras, Jamaica, Mexico, Nicaragua, Panama, Paraguay, Peru, Saint Kitts and Nevis, Saint Lucia, Saint Maarten, Trinidad and Tobago, Uruguay, Venezuela

**Middle East (16 countries)**

Bahrain, Iran, Iraq, Israel, Jordan, Kurdistan, Kuwait, Lebanon, Oman, Palestine, Qatar,

Saudi Arabia, Syria, Turkey, United Arab Emirates, Yemen

**North America (3 countries)**

Canada, Puerto Rico, United States
